# Blue light irradiation inhibits the M2 polarization of the cancer-associated macrophages in colon cancer

**DOI:** 10.1186/s12885-024-12440-1

**Published:** 2024-05-31

**Authors:** Toshiaki Yoshimoto, Masaaki Nishi, Shohei Okikawa, Kozo Yoshikawa, Takuya Tokunaga, Toshihiro Nakao, Chie Takasu, Hideya Kashihara, Yuma Wada, Takayuki Noma, Mitsuo Shimada

**Affiliations:** Department of Surgery, Tokushima University Graduate School, 3-18-15 Kuramoto-cho, Tokushima City, 770-8503 Tokushima, Japan

**Keywords:** Blue light, Colorectal cancer, Light-emitting diode, Opsin3, Tumor-associated macrophage

## Abstract

Recent studies have shown that blue light-emitting diode (LED) light has anti-tumor effects, suggesting the possibility of using visible light in cancer therapy. However, the effects of blue light irradiation on cells in the tumor microenvironment, including tumor-associated macrophages (TAMs), are unknown. Here, THP-1 cells were cultured in the conditioned medium (CM) of HCT-116 cells to prepare TAMs. TAMs were divided into LED-irradiated and control groups. Then, the effects of blue LED irradiation on TAM activation were examined. Expression levels of M2 macrophage markers CD163 and CD206 expression were significantly decreased in LED-irradiated TAMs compared with the control group. While control TAM-CM could induce HCT-116 cell migration, these effects were not observed in cells cultured in TAM-CM with LED irradiation. Vascular endothelial growth factor (VEGF) secretion was significantly suppressed in LED-exposed TAMs. PD-L1 expression was upregulated in HCT-116 cells cultured with TAM-CM but attenuated in cells cultured with LED-irradiated TAM-CM. In an in vivo model, protein expression levels of F4/80 and CD163, which are TAM markers, were reduced in the LED-exposed group. These results indicate that blue LED light may have an inhibitory effect on TAMs, as well as anti-tumor effects on colon cancer cells.

## Introduction

Irradiating cells and living organisms with light-emitting diodes (LEDs) with specific wavelengths of light has a variety of effects. Photodynamic therapy (PDT), which combines light and photosensitizers, is attracting attention as a new minimally invasive alternative to chemoradiotherapy. PDT is being clinically applied to treat esophageal cancer, lung cancer, brain tumors, and other cancer types [1]. Several in vitro and in vivo reports have also demonstrated the efficacy of PDT for colorectal cancer [2, 3]. However, recent studies have demonstrated that using blue LED light alone is effective for suppressing the growth of some cancer cell types. Specific mechanisms for the phototoxic and anti-proliferative effects of blue light have been reported, including the induction of autophagy [4], necroptosis [5], apoptosis [6], and cell cycle arrest [7]. Blue light irradiation can also result in the production of reactive oxygen species and DNA damage in colon cancer cells [8]. In a previous report, we showed that using 465 nm blue light irradiation (30 mW/cm^2^, 30 min) could inhibit colon cancer cell growth via inducing autophagy [9]. Furthermore, we revealed that the blue photoreceptor Opsin3 (Opn3), which is expressed in colon cancer cells, can promote the cytotoxic effects of blue light.

The opsin family of proteins is composed of G-protein-coupled receptors (GPCRs) that are expressed on photoreceptor cells in the retina. Opn3 regulates GPCR signaling and acts as a receptor for blue light [10]. Additionally, Opn3 activates the G_i/o_ subtype of G proteins with light at a wavelength of 460 to 470 nm, inhibits adenylate cyclase, and decreases cyclic adenosine monophosphate (cAMP) [10, 11]. Recent studies have shown that Opn3 is also widely expressed in organs unrelated to vision, such as the respiratory epithelium, liver, kidney, and heart, and is called non-visual opsin [12, 13]. One report indicates that Opn3 acts as a photoreceptor in malignant melanocytes and contributes to phototherapy [14], and our findings indicate that Opn3 is a potential target for phototherapy as a photoreceptor in colon cancer cells [9].

Regulation of the tumor microenvironment (TME), which is composed of various cell types including fibroblasts, immune cells, and tumor cells, is crucial for cancer therapy. We recently reported that irradiation of cancer-associated fibroblasts (CAFs) with blue light can decrease CAF activity and interleukin (IL)-6 secretion, which can support decreased tumor malignancy of colon cancer cells [15]. There is a report regarding tumor-associated macrophages (TAM), a TME component other than CAFs, indicating that PDT leads to a decrease in M2 macrophages and an increase in M1 macrophages [16]. TAM is also an important therapeutic target; however, the impact of blue light on TAM remains unclear. Additionally, there are no reports on the blue light receptors of TAM.

In this study, we focused on TAMs, mainly M2 macrophages, which are involved in tumor growth and metastasis through the secretion of cytokines, such as VEGF and PD-L1. We investigated the impact of blue LED irradiation on TAMs to examine its effects on the TME.

## Materials and methods

### Cell culture

The human colon cancer cell line HCT-116 (KAC Co., Ltd., Kyoto, Japan, Cat. No.: EC91091005-F0) was used for in vitro and in vivo experiments. The cell line was confirmed to be negative for mycoplasma. Cells were maintained and cultured in accordance with international guidelines for proper cell culture practices. Cells were cultured in RPMI 1640 medium (Wako, Osaka, Japan) supplemented with 10% fetal bovine serum (FBS), 100 U/mL penicillin, and 100 µg/mL streptomycin (Sigma-Aldrich, St. Louis, MO, USA) in a 37 °C humidified incubator with 5% CO_2_.

### Preparation of conditioned medium (CM) and TAMs

To obtain CM, HCT-116 cells were cultured in 100 mm culture dishes until 80% confluent. The cells were washed twice with pre-warmed phosphate-buffered saline (PBS) and incubated in fresh medium without FBS. After incubation at 37 °C for 48 h, the supernatant was collected, centrifuged at 800×g for 5 min at 22 °C, and filtered through a 0.2-µm sterile filter.

To obtain TAMs, THP-1 cells were exposed to 150nM PMA for 48 h to induce differentiation into M0 macrophages, then they were treated with CM derived from HCT-116 cells. CM was added to the culture medium at a 1:1 ratio and cells were incubated at 37 °C for 48 h.

**Fig. 1:**
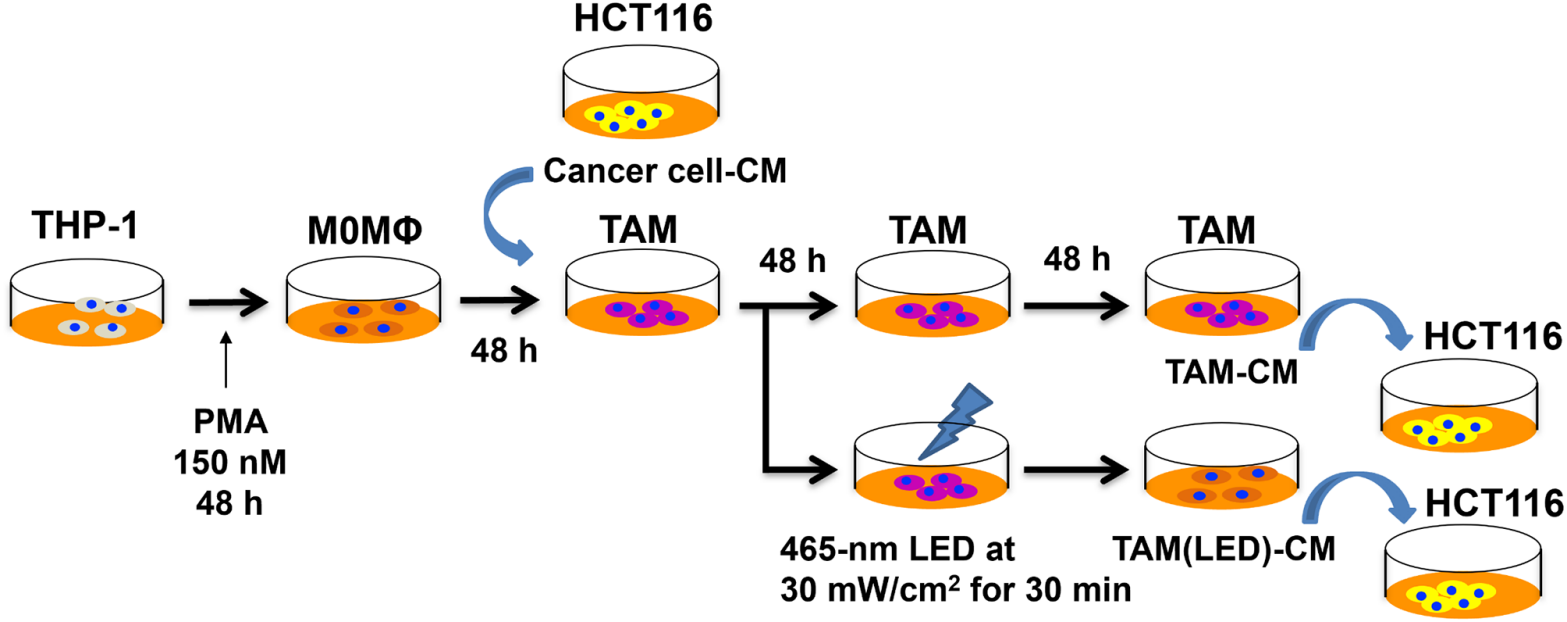
Procedures for the generation of tumor-associated macrophages (TAMs), conditioned medium (CM), and irradiation. M0 macrophages (from PMA-treated THP-1 cells) were cultured in the CM of HCT-116 colon cancer cells to generate TAMs. The TAMs were either untreated (control group) or irradiated as indicated with blue light. The medium was changed once, and the supernatant (TAM-CM) was collected. The TAM-CM from the irradiated and control groups was used for subsequent experiments

To obtain TAM-CM, the TAMs were treated with blue LED light (465 nm, 30 mW/cm^2^, 30 min) (irradiation group) or left untreated. The medium was changed once and the supernatant (TAM-CM) was collected. A schematic for these processes is shown in Fig. 1.

### Animals

Four-week-old female BALB/c mice were purchased from Charles River Japan, Inc. (Kanagawa, Japan). Animals were caged under controlled conditions and provided water and standard laboratory rations for at least seven days prior to use. Before and after surgery, the animals had free access to tap water and food. The experiments and procedures in this study were approved by the Animal Welfare and Use Committee of the University of Tokushima (Approval No. T2021-42). All animal experiments were performed in accordance with the U.K. Animals (Scientific Procedures) Act 1986 and associated guidelines, the EU Directive 2010/63/EU for animal experiments, and the National Institutes of Health Guide for the Care and Use of Laboratory Animals (NIH Publications No. 8023, revised 1978).

### Orthotopic model and LED irradiation

CT-26 cells (ATCC, VA, USA, Cat. No.: CRL-2638) were harvested with 1 mM ethylenediaminetetraacetic acid (EDTA) in PBS, washed three times with PBS, and resuspended at a density of 1 × 10^7^ cells/mL in PBS containing 500 mg/mL of Matrigel (Becton Dickinson Labware, Bedford, MA, USA). Animals (*n* = 8) were anesthetized with isoflurane (3–4% for induction and 1–2% for maintenance). A 7-mm incision was made in the anterior rectal wall to prevent colonic obstruction caused by rectal tumor progression. CT-26 cells (1 × 10^6^) were injected into the submucosa of the posterior wall of the rectum using a 29-gauge needle.

Seven days after cell implantation, the mice developed rectal cancer. The mice were randomized into two groups (*n* = 4 mice per group) and treated as follows: (i) the LED irradiation group (465 nm, 30 mW/cm^2^, 30 min, once) and (ii) the control group (untreated). The mice were sacrificed by cervical spinal cord dissection at 2 weeks after cell implantation. A humane endpoint was set at the stage when the tumor size increased by more than 10% of the mice’s body weight, but this did not apply to any of the mice.

A 465-nm blue LED (NCSB119, NICHIA Corporation, Tokushima, Japan) was used as a light source. An LED irradiation device (Department of Electrical and Electronic Engineering, Faculty of Engineering, The University of Tokushima, Tokushima, Japan) was used for irradiation experiments.

### Immunohistochemistry (IHC)

The tissues resected from the orthotopic model or cultured cells were fixed in 10% formalin (Wako 066-03847) for 24 h at room temperature, paraffin-embedded, and sliced into 4-µm-thick sections. The samples were blocked in 3% BSA (MACS^®^ BSA Stock Solution 130-091-376) for 1 h at room temperature. An anti-F4/80 antibody (15361-1-AP; Proteintech, Rosemont, IL, USA), anti-CD163 antibody (SAB2700986; Sigma-Aldrich), and anti-PD-L1 antibody (17952-1-AP; Proteintech) were used for IHC staining following the manufacturer’s instructions. The fluorescence area was measured using Image J software (ver. 1.53, National Institutes of Health, Bethesda, MD, USA).

### Quantitative real-time PCR (qRT-PCR) analysis

Total RNA was isolated from samples using the RNeasy Mini Kit (Qiagen, Hilden, Germany) and reverse transcribed using a high-capacity cDNA reverse transcription kit (Applied Biosystems, Tokyo, Japan). Then, qRT-PCR was performed using a 7500 real-time PCR system, TaqMan gene expression assay on demand, and TaqMan Universal Master Mix (Applied Biosystems). The following TaqMan assays were used: Opn3 (Hs00173892_m1), CD163 (Hs00174705_m1), CD206 (Hs00267207_m1), and PD-L1 (Hs00204257_m1). GAPDH (4326317E) was used as an internal control for mRNA expression. The thermal cycler conditions were as follows: 2 min at 50 °C, 10 min at 95 °C, and then 40 cycles of 15 s at 95 °C and 1 min at 60 °C. Amplification data were analyzed using the Prism 7500 Sequence Detection System ver. 1.3.1 (Applied Biosystems). qRT-PCR was performed using a Step One Plus Real-Time PCR System (Applied Biosystems). The relative target transcript expression levels were assessed and normalized to the expression levels of GAPDH mRNA as an internal control using the 2^−ΔΔCq^ method [17].

### Migration and scratch assays

For migration assays, Transwell inserts (Corning, Corning, NY, USA) with a pore size of 8 μm were used. CT-116 cells (2 × 10^4^) were seeded into the upper chamber. After cell attachment, the medium was discarded and fresh medium containing 1% FBS was added to the upper chamber. CM containing 10% FBS was added to the lower chamber. After incubation for 24 h, cells at the bottom of the Transwell inserts were fixed in 4% paraformaldehyde, then stained with 0.2% crystal violet. The stained cells in three random microscopic fields (100×) were counted.

For scratch assays, HCT-116 cells were seeded at a density of 2 × 10^4^ cells/well in 6-well plates. After the cells became 100% confluent, a plastic pipette tip was used to scrape the center of the well, creating a 1-mm-wide scratch. The medium was discarded and fresh DMEM containing 1% FBS was added, followed by CM at a 1:1 ratio. The final FBS concentration was 0.5%. The cells were then incubated at 37 °C for 24 h. Images of the wound were taken using a phase-contrast microscope (40× magnification; DP22-CU; Olympus Corporation) at 0 and 24 h after scratching.

### Statistical analysis

All statistical analyses were performed using Stat View version 5.0 software (SAS Institute, Cary, NC, USA). The Mann-Whitney U-test and Kruskal-Wallis test followed by Bonferroni Mann-Whitney U test were used for statistical comparisons. *P*-values less than 0.05 were considered statistically significant.

## Results

### Opn3 expression in M0 macrophages and TAMs

**Fig. 2:**
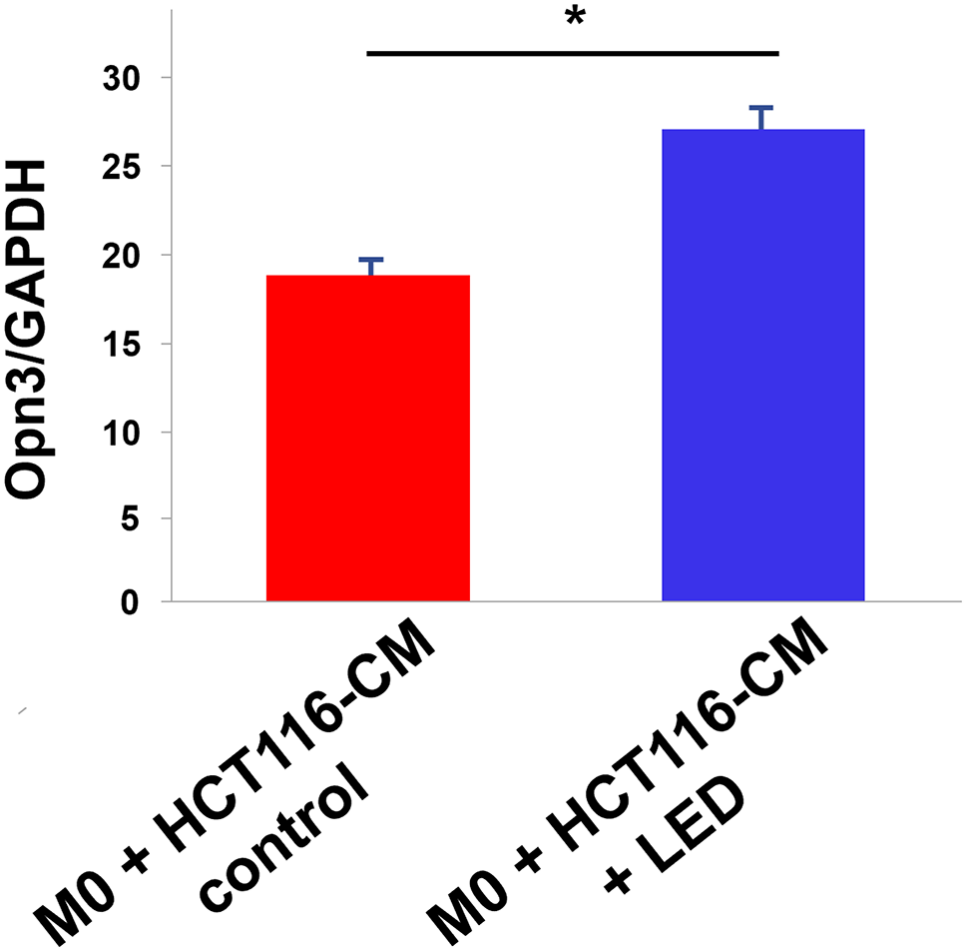
Opsin 3 (Opn3) mRNA expression levels in M0 macrophages and tumor-associated macrophages (TAMs). Opn3 mRNA expression levels were examined in TAMs with or without blue LED irradiation

We examined Opn3 mRNA expression levels in TAMs generated by culturing M0 macrophages with the CM of HCT-116 colon cancer cells, which were then exposed to LED or left untreated. LED-irradiated TAMs showed higher Opn3 expression levels compared with the untreated control group (*P* < 0.05; Fig. 2).

### Blue LED irradiation suppressed the polarization of macrophages

**Fig. 3:**
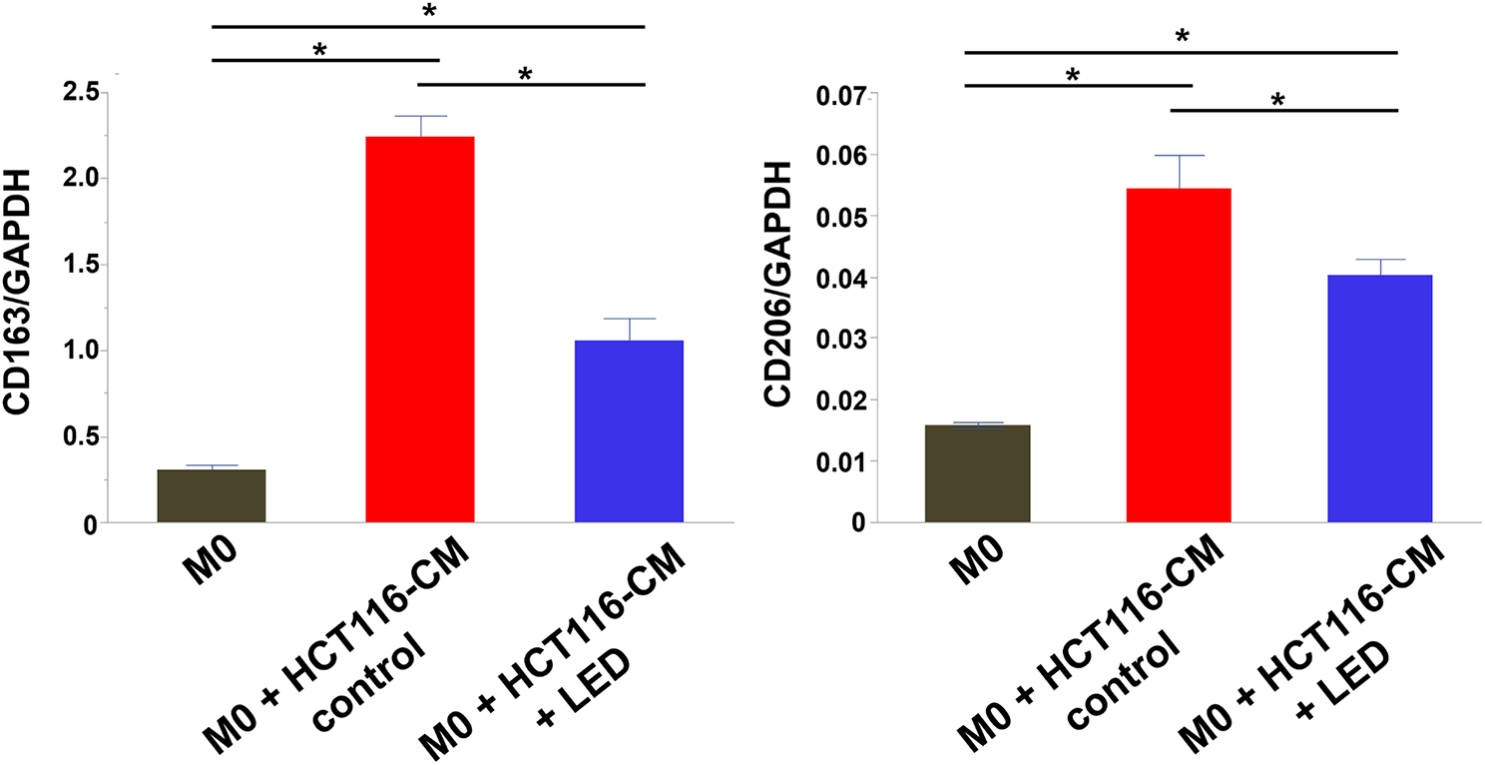
Effects on blue LED irradiation on tumor-associated macrophage (TAM) polarization. Expression levels of CD163 and CD206 were examined in M0 macrophages, TAMs, and blue LED-irradiated TAMs

The mRNA expression levels of M2 macrophage markers CD163 and CD206 were significantly upregulated in TAMs (M0 macrophages cultured in HCT-116 cell CM) compared with those of M0 macrophages (*P* < 0.05; Fig. 3). In contrast, CD163 and CD206 mRNA expression levels were decreased in TAMs treated with blue LED light irradiation, suggesting that blue LED light could inhibit the polarity change to M2 macrophages (*P* < 0.05; Fig. 3).

### Migration and scratch assays for HCT-116 cells cultured in TAM-CM

**Fig. 4:**
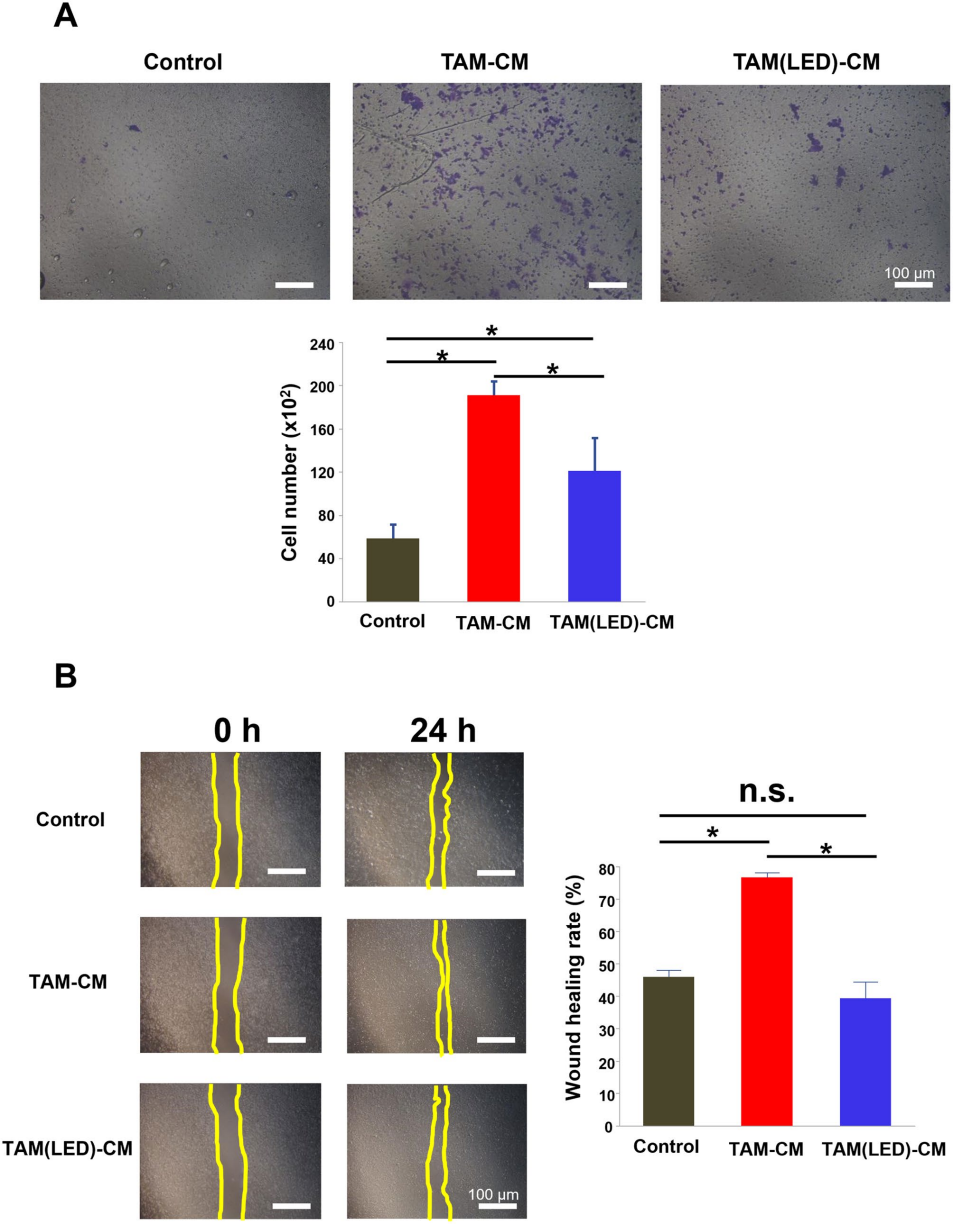
Migration and scratch assays for HCT-116 cells cultured in tumor-associated macrophage conditioned medium (TAM-CM). (**A**) Migration assay results for HCT-116 cells cultured in TAM-CM from untreated or blue LED-irradiated TAMs. (**B**) Scratch assay results for HCT-116 cells cultured in TAM-CM from untreated or blue LED-irradiated TAMs

HCT-116 cells were cultured in TAM-CM, then migration (Fig. 4A) and scratch assays (Fig. 4B) were performed. Culturing the cells in TAM-CM significantly promoted the migration of HCT-116 cells (*P* < 0.05). However, TAM-CM derived from TAMs irradiated with blue light did not promote the migration of HCT-116 cells. In scratch assays, HCT-116 cells cultured in TAM-CM increased wound healing rates (*P* < 0.05), whereas TAM-CM derived from TAMs irradiated with blue light did not increase the wound healing rate of HCT-116 cells.

### Blue LED irradiation suppressed TAM secretion of VEGF

**Fig. 5:**
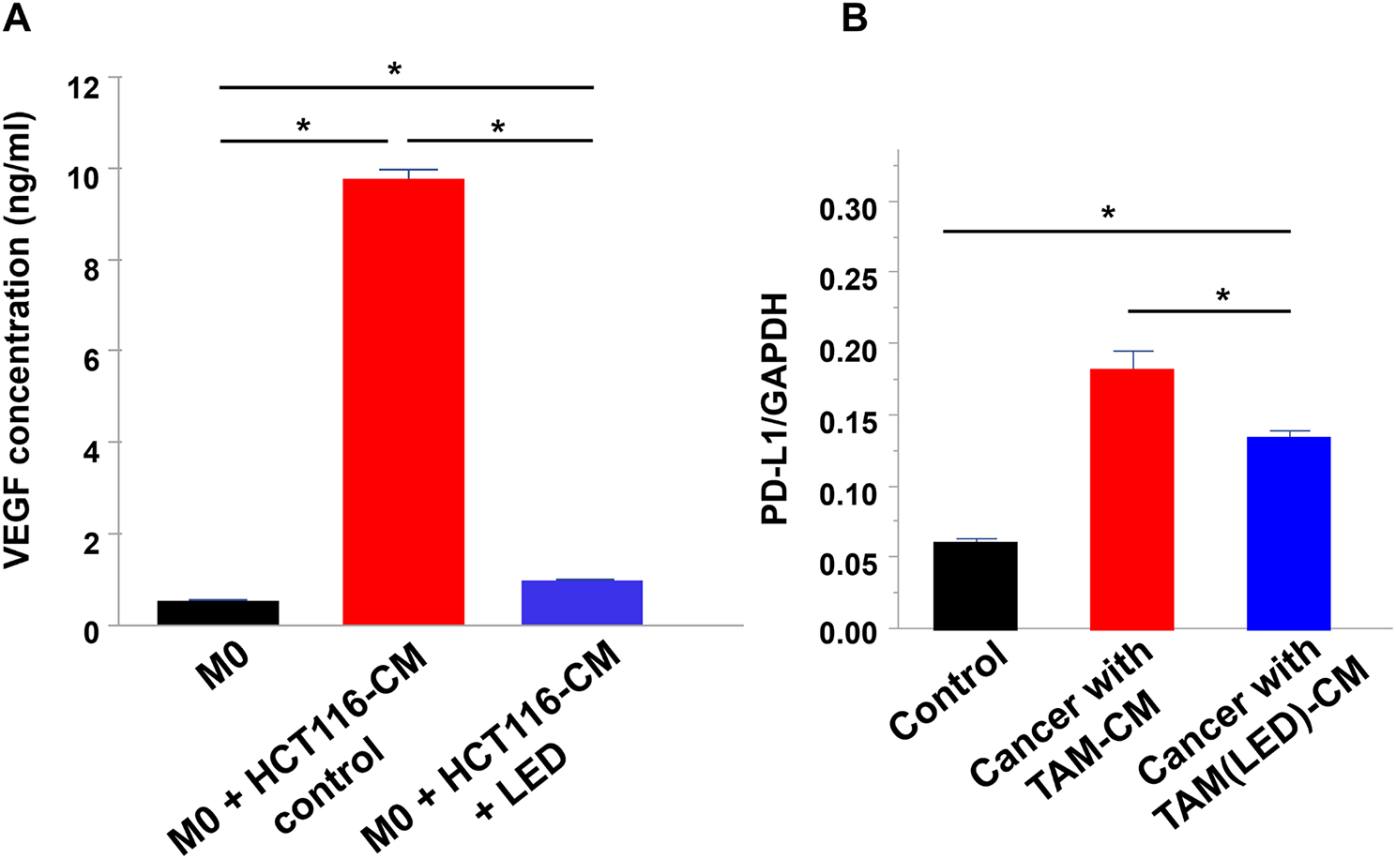
Vascular endothelial growth factor (VEGF) secretion by tumor-associated macrophages (TAMs) and PD-L1 expression in HCT-116 cells cultured in TAM-CM. (**A**)Levels of VEGF secretion were measured in M0 macrophages, TAMs, and blue LED-irradiated TAMs. (**B**) PD-L1 expression levels were measured in HCT-116 cells cultured in TAM-CM from untreated or blue LED-irradiated TAMs

As shown in Fig. 5A, the levels of VEGF secretion were significantly higher in TAMs compared with M0 macrophages (*P* < 0.05). However, in LED-irradiated TAMs, VEGF secretion was significantly suppressed (*P* < 0.05).

### PD-L1 expression in HCT-116 cells cultured in TAM-CM

In HCT-116 cells cultured with TAM-CM, PD-L1 mRNA expression levels were upregulated (*P* < 0.05; Fig. 5B). However, the degree of PD-L1 mRNA expression induction was attenuated in HCT-116 cells cultured with TAM-CM and irradiated with blue LED (*P* < 0.05; Fig. 5B).

### Effects of blue LED irradiation in an in vivo model

**Fig. 6:**
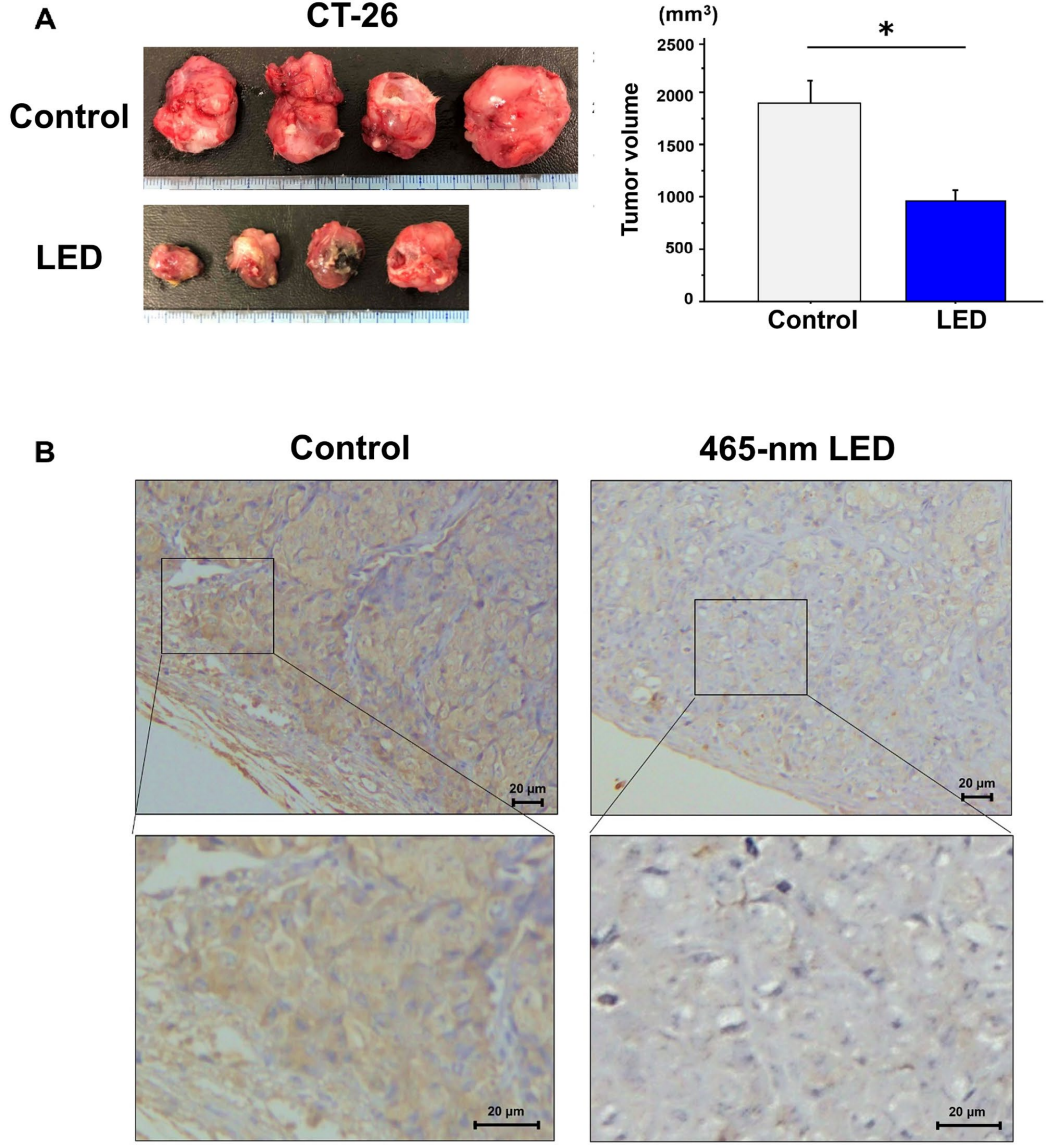
Tumor suppressive effects of blue LED irradiation in an orthotopic model. (**A**) The in vivo models were irradiated with blue LED once a week and tumor size was measured 2 weeks after tumor injection. (**B**) Immunohistochemistry assays showed attenuated PD-L1 protein expression in LED-irradiated tumors

**Fig. 7:**
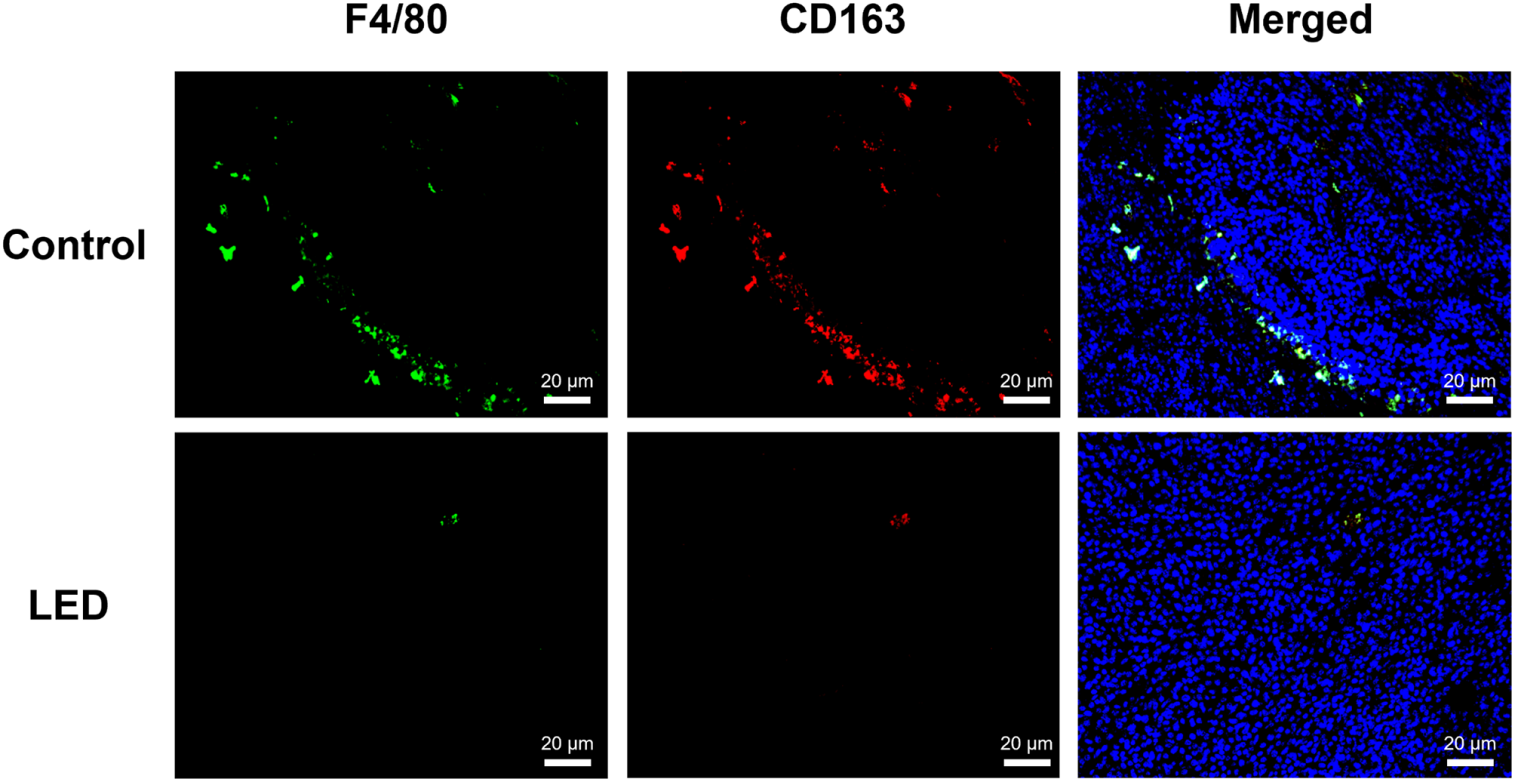
Immunofluorescence staining (IF) results of tumor-associated macrophage (TAM) marker expression in the in vivo model. IF assays were used to examine cells that were positive for F4/80 or CD163 protein expression within tumors

Colon cancer tumor growth in a mouse model was significantly suppressed by blue LED irradiation (*P* < 0.05; Fig. 6A), as indicated by reduced tumor size. PD-L1 expression levels were downregulated in tumors exposed to blue LED irradiation (Fig. 6B). Furthermore, immunofluorescence staining (IF) showed that tumors irradiated with blue LED showed fewer cells positive for F4/80 and CD163 protein compared with the control samples (Fig. 7). The CD163-positive cell areas in five randomly selected fields of view were compared, with a significant reduction observed in the irradiated group compared with the control group (*P* < 0.05).

## Discussion

Recent studies have demonstrated that cells in the TME, including fibroblasts and immune cells, are extremely important for the efficacy of anti-cancer therapies. We previously reported that blue LED light can act in an inhibitory manner on not only tumor cells, but also on CAFs, resulting in reduced tumor malignancy [15]. In the present study, we examined the impact of blue LED on the TME by investigating its effects on TAMs.

Our results showed that blue light irradiation could decrease the expression of M2 macrophage markers CD163 and CD206, suggesting that it could suppress macrophage polarization to the M2 phenotype. In addition, migration assays showed that HCT-116 cell migration was promoted in the presence of CM from non-LED-irradiated TAMs but was significantly reduced with CM from LED-irradiated TAMs.

We previously reported that VEGF secretion by TAMs plays a significant role in tumor malignancy in hepatocellular carcinoma [18]. In the present study, VEGF secretion was observed in TAMs without LED irradiation, but this was significantly suppressed in TAMs irradiated with LED. In addition, HCT-116 cells cultured in TAM-CM showed elevated PD-L1 expression levels, whereas cells cultured with LED-irradiated TAM-CM showed attenuated induction of PD-L1 expression. The transcription factor STAT3 can reportedly regulate expression of the M2 macrophage markers CD163 and CD206 [19]. Additionally, STAT3 has been implicated in elevated tumor malignancy and PD-L1 expression through VEGF in tumor cells [20]. Furthermore, it is reported that that the downstream signal of G-protein-coupled receptors, the cAMP pathway, controls polarization towards M2 macrophages [21]. In this experiment, it is possible that the inhibition of adenylate cyclase by the Gi/o subtype of G proteins due to blue light irradiation led to a decrease in cAMP, potentially resulting in the suppression of polarization towards M2 macrophages.

Recently, PDT targeting of TAMs has been reported [16]. We have also reported the effect of blue LEDs on CAFs, suggesting the future possibility of phototherapy targeting the TME. Furthermore, OPN3 is expressed in TAMs, suggesting the possibility that blue LEDs may suppress TAMs via photoreceptors.

In our previous experiments, similar anti-tumor effects were observed with daily 5-minute irradiation and a single 30-minute irradiation [9]. Therefore, we used a 30-minute irradiation in the current study. When M0 and M2 macrophages were irradiated with blue light for 30 min, no decrease in cell viability was observed (data not shown). Subsequent investigations should focus on evaluating the impact of short-duration fractionated irradiation on TAMs, as well as the effects of frequent 30-minute irradiation sessions. In the future, it will be necessary to investigate the effects of blue light on other M2 macrophage markers such as IL-10, IL-4, and IL-13, as well as its impact on M1 macrophages.

The major limitations of this study are that experiments using mouse colorectal cancer cells and mouse macrophages were not conducted and that the significance of OPN3 expression in tumors and macrophages has not been fully investigated. Future studies involving OPN3 expression knocked down in macrophages or with OPN3 knockout mice are required. Additionally, more quantitative experiments such as flow cytometry need to be conducted. Although further studies are required to fully elucidate the mechanism of action of blue LED irradiation on tumors and the TME, this study demonstrated significant effects of blue LED irradiation on colon cancer cells and TAMs.

**Fig. 8:**
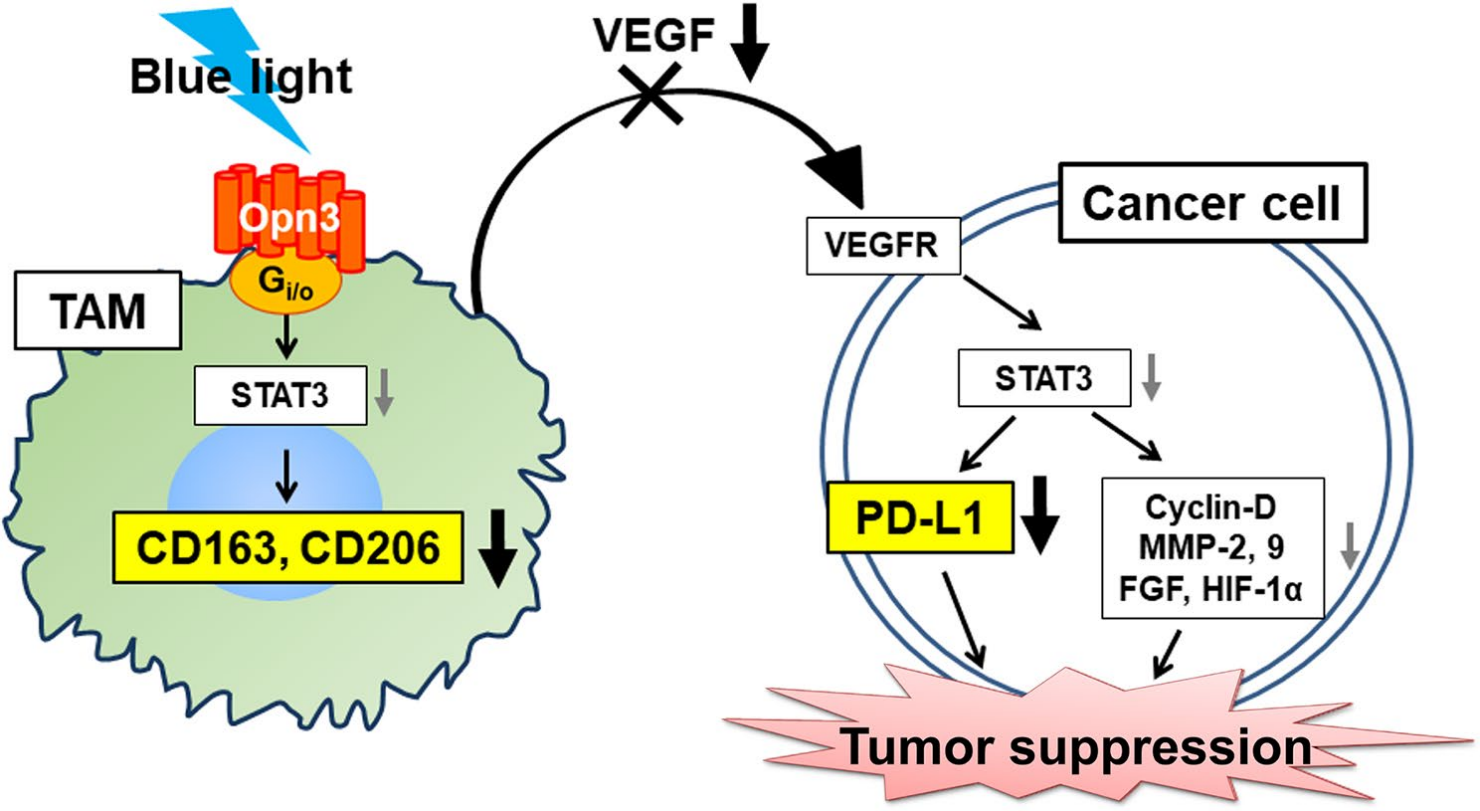
Summarized mechanisms of the effects of blue LED irradiation on tumor-associated macrophages (TAMs)

In summary, blue light could suppress macrophage polarization to TAMs and decreased the levels of VEGF secretion. Additionally, blue light attenuated TAM-mediated tumor malignancy and decreased PD-L1 expression levels. The blue light receptor OPN3 is expressed in TAMs, and macrophage polarization and activity were reduced by blue light irradiation. Taken together, these findings suggest that blue light may regulate TAMs in tumors and the TME (Fig. 8).

## Data Availability

The authors declare that the data supporting the findings of this study are available within the paper. Should any raw data files be needed in another format they are available from the corresponding author upon reasonable request. Source data are provided with this paper.

## Abbreviations

LED: Light-Emitting Diode
TAM: Tumor-Associated Macrophage
CM: Conditioned Medium
VEGF: Vascular Endothelial Growth Factor
Opn3: Opsin 3
CAF: Cancer-Associated Fibroblast
cAMP: Cyclic Adenosine Monophosphate
GPCR: G Protein-Coupled Receptor
PDT: Photodynamic Therapy
IHC: Immune Histo Chemistry
IF: Immune Fluorescence Staining
